# Sphetcher: Spherical Thresholding Improves Sketching of Single-Cell Transcriptomic Heterogeneity

**DOI:** 10.1016/j.isci.2020.101126

**Published:** 2020-05-04

**Authors:** Van Hoan Do, Khaled Elbassioni, Stefan Canzar

**Affiliations:** 1 Gene Center, Ludwig-Maximilians-Universität München, 81377 Munich, Germany; 2 Khalifa University of Science and Technology, P.O. Box: 127788, Abu Dhabi, UAE

**Keywords:** Bioinformatics, Transcriptomics, Data Analysis

## Abstract

The massive size of single-cell RNA sequencing datasets often exceeds the capability of current computational analysis methods to solve routine tasks such as detection of cell types. Recently, geometric sketching was introduced as an alternative to uniform subsampling. It selects a subset of cells (the sketch) that evenly cover the transcriptomic space occupied by the original dataset, to accelerate downstream analyses and highlight rare cell types. Here, we propose algorithm Sphetcher that makes use of the thresholding technique to efficiently pick representative cells within spheres (as opposed to the typically used equal-sized boxes) that cover the entire transcriptomic space. We show that the spherical sketch computed by Sphetcher constitutes a more accurate representation of the original transcriptomic landscape. Our optimization scheme allows to include fairness aspects that can encode prior biological or experimental knowledge. We show how a fair sampling can inform the inference of the trajectory of human skeletal muscle myoblast differentiation.

## Highlights

• Sphetcher distils large-scale scRNA-seq data down to a small selection of cells
• Spheres of small radius around selected cells cover the original transcriptomic space
• Selection enhances and accelerates downstream analysis such as trajectory inference
• Sphetcher can leverage existing annotation of known cell types

## Graphical Abstract

**Figure:**
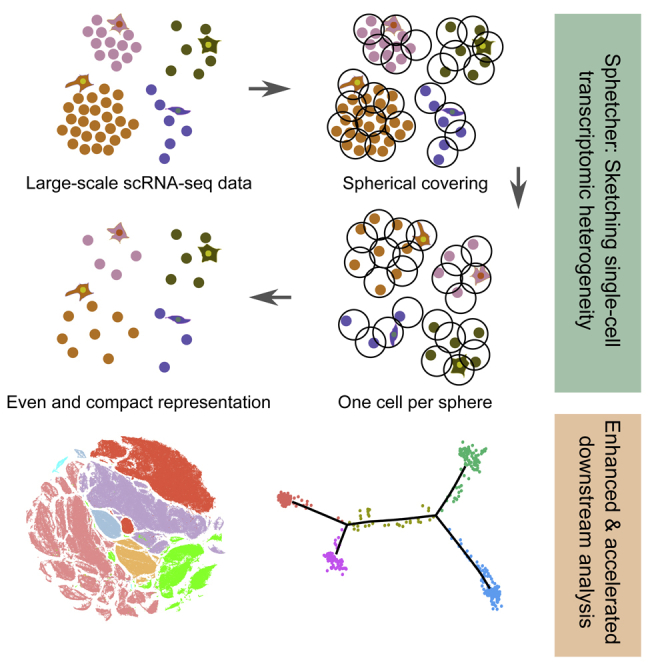

## Introduction

Single-cell RNA sequencing (scRNA-seq) has emerged as a revolutionary tool that can shed light on many corners of cell biology that were unaccessible to previous approaches. The technology has improved dramatically over the last few years, especially in terms of throughput. Droplet-based technologies allow to profile the expression of every gene in the genome for hundreds of thousands of cells at once, and even experiments profiling the transcriptome of millions of cells have become increasingly common (Cao et al., 2019). Furthermore, the meaningful interpretation of single-cell datasets requires their integration across different biological contexts, yielding datasets whose enormous size exceeds the capability of current computational analysis methods to solve routine tasks such as clustering, trajectory inference, and visualization in practical time or require excessive amounts of memory.

In practice, methods are often run on a smaller subset of the data to bridge the gap between the scalability of the algorithm and the volume of the data (Hie et al., 2019). The commonly applied uniform subsampling strategy, however, ignores the similarity or dissimilarity between gene expression patterns of single cells and thus risks overlooking rare cell states. Spatial random sampling (SRS) (Rahmani and Atia, 2017) and *k*-means++ (Arthur and Vassilvitskii, 2007), on the other hand, take into account the structure of the data when sampling the data. Experiments performed in Hie et al. (2019), however, demonstrated that these data-dependent methods do not scale efficiently to large datasets and provide unbalanced samples that hamper downstream analyses. Clustering the full data first followed by sampling from clusters, as performed by dropClust (Sinha et al., 2018), has similar issues (Hie et al., 2019). Hie et al. (2019) introduced *geometric sketching* as an alternative approach that efficiently samples cells evenly across gene expression space rather than proportional to the abundance of cells that are in a similar state. For purely computational reasons, however, Hie et al. (2019) approximate the transcriptomic space of single cells by equal-sized boxes rather than spheres, from within which cells are randomly selected as representatives into the *sketch*.

Here, we propose algorithm Sphetcher that makes use of the thresholding technique originally proposed for the design of approximation algorithms for bottleneck problems to efficiently pick representative cells within spheres of a fixed size into a *spherical sketch* of different metric spaces. We provide theoretical guarantees for the spherical sketch computed by Sphetcher and demonstrate through experiments on six single-cell datasets that these theoretical guarantees are indeed reflected in a more accurate representation of the original transcriptomic space, which in turn benefits downstream analyses such as clustering and allows to detect a rare population of inflammatory macrophages. Furthermore, our optimization scheme naturally allows to include fairness aspects that require to include cells of each pre-defined category that can encode prior biological or experimental knowledge such as cell type or collection time point. We demonstrate how our fairness-inspired model can help to incorporate the collection time point of cells in a time series experiment into the reconstruction of their developmental trajectory. Carefully combined with a prior grid sampling strategy that is orders of magnitude faster than geometric sketching, Sphetcher requires only 16 minutes to compute a sketch for a mouse embryonic dataset comprising two million cells.

## Results

### Overview of Our Spherical Sketching Algorithm

Given a large scRNA-seq dataset, we seek to select a subset of cells, a so-called *sketch* (Hie et al., 2019), that evenly represents the geometry of the transcriptional space occupied by the original data. As originally proposed in Hie et al. (2019), we intuitively aim at capturing the transcriptional heterogeneity of single cells by removing predominantly cells that show similar expression patterns to other cells while preserving rare cell states. A sketch of a given size represents the full data well if every original cell is close to a cell in the sketch, according to some measure of distance between two cells. In other words, spheres of a small radius centered at each cell in the sketch must contain, or *cover*, every cell in the full dataset. The smaller the radius, the better the sketch represents the original transcriptional space.

**Figure 1:**
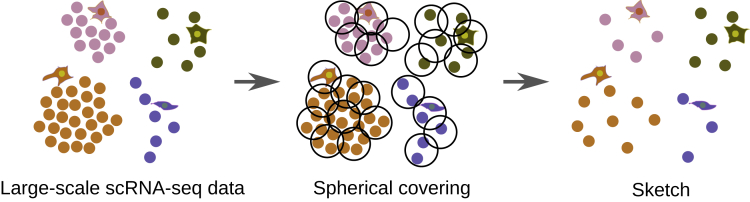
Overview of Sphetcher For a (large) scRNA-seq dataset (left), Sphetcher uses a disk-friendly greedy algorithm to compute a smallest size set of spheres of a fixed radius that cover all cells (middle). It guesses the smallest possible radius such that a given number of spheres of that radius suffice to cover all cells. One representative cell (the center) from each sphere is selected into the final spherical sketch (right).

Our algorithm implemented in software tool Sphetcher guesses the smallest possible radius for which a sketch of a given size exists that covers all remaining cells with spheres of this radius (Figure 1). For each guess, it computes the smallest size sketch that covers all cells and tries a smaller or larger radius in the next iteration if the resulting sketch contains too few or too many cells, respectively. It computes the smallest sketch that covers all cells using a greedy set cover approach. In each iteration it adds the cell to the sketch that contains the largest number of yet uncovered cells within the given distance. We employ the disk-friendly greedy (DFG) algorithm developed in Cormode et al. (2010) that scales to very large scRNA-seq datasets. For very large datasets, the spherical sketching approach is combined with a prior grid sampling that we show increases the radius of covering spheres by only a small factor (Transparent Methods).

In addition, our greedy algorithm can incorporate prior categorical information on, e.g., biological cell types or collection time point of cells. In a *fairness*-inspired model it selects at least a given number of representatives from each class into the sketch.

A detailed description of our algorithm and the parameters used in the experiments are provided in Transparent Methods. We also provide a theoretical analysis that shows that if we are willing to include slightly more cells in the sketch, our greedy algorithm is guaranteed to find the covering of cells with spheres with optimal, that is, with smallest possible radius. Furthermore, we give theoretical justification for the practical performance of our greedy set cover approach and its robustness to noise present in scRNA-seq data.

### Sphetcher More Accurately Sketches the Transcriptomic Space

**Figure 2:**
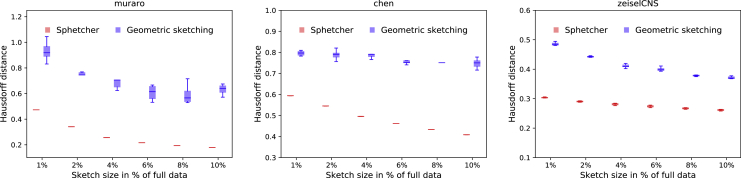
Comparison of Hausdorff Distances The spherical sketch computed by Sphetcher exhibits consistently smaller Hausdorff distances to the full dataset than geometric sketching, across datasets and sketch sizes. For each sketch size, the results of 10 random trials are shown. Results on datasets zeisel, klein, and saunders are shown in Figure S1. Figure S3 shows Hausdorff distances achieved by our naïve grid sampling strategy on datasets zeiselCNS and saunders.

To evaluate how well the *spherical sketch* computed by our method Sphetcher represents the original transcriptomic space, we use the same robust Hausdorff distance measure as Hie et al. (2019) (Transparent Methods). Intuitively, a small Hausdorff distance between a sketch and a full dataset indicates an accurate representation that contains for every cell in the original data a close cell in the sketch. We compare our sketch to the *geometric sketch* computed by Hie et al. (2019), which the authors demonstrated to consistently achieve smaller Hausdorff distances than uniform sampling and data-dependent sampling methods SRS and *k*-means++. The geometric sketch computed in Hie et al. (2019) seeks to minimize the same objective function (Transparent Methods) but simplifies the approximation of the geometric space by equal-sized boxes rather than spheres. We benchmark Sphetcher on six public single-cell datasets from mouse and human that vary in size and number of cell populations: human pancreas (*muraro*) (Muraro et al., 2016) with 2,126 cells, 10 populations; mouse embryonic stem cells (*klein*) (Klein et al., 2015) with 2,717 cells, 4 populations; mouse cortex and hippocampus (*zeisel*) (Zeisel et al., 2015) with 3,005 cells, 9 populations; mouse hypothalamus (*chen*) (Chen et al., 2017) with 14,437 cells, 47 populations; mouse nervous system (*zeiselCNS*) (Zeisel et al., 2018) with 465,281 cells, 7 populations; and adult mouse brain (*saunders*) (Saunders et al., 2018) with 665,858 cells and 11 populations. Figures 2 and S1 show the Hausdorff distances of 10 random trials on sketch sizes ranging from 1% to 10% of the full dataset. Values reported here can deviate slightly from the original publication (Hie et al., 2019) due to different preprocessing (Transparent Methods). Our sampling approach based on spheres results in sketches that consistently lead to smaller Hausdorff distances, across datasets and sketch sizes. As expected, larger sketches yield smaller Hausdorff distances, but across all datasets the geometric sketch based on 10% of the data does not represent the full data as well as our spherical sketch with just 1% of the data. In addition, sketches computed by Sphetcher exhibit a considerably smaller variability over the random trials (Figure S2). Although the geometric sketch randomly picks a cell in each box, Sphetcher's only random decision is in breaking ties between equal-sized sets during the greedy set cover computation (Transparent Methods). Remarkably, our naive grid sampling strategy alone, which is part of our hybrid alternative for very large datasets (Transparent Methods), achieves competitive Hausdorff distances on datasets zeiselCNS and saunders, especially for small sketch sizes (Figure S3).

### Clustering of Spherical Sketches Facilitates Cell-Type Identification

**Figure 3:**
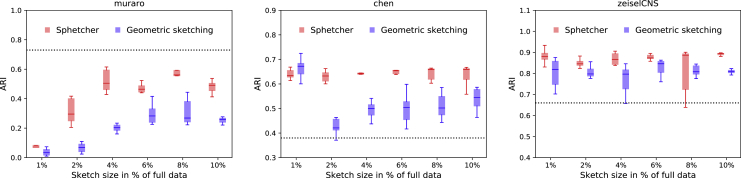
Comparison of Sketch-Based Clustering Accuracy Louvain clustering of spherical sketches computed by Sphetcher yields more accurate cell clusterings as measured by Adjusted Rand Index (ARI) than geometric sketching based clustering. In both cases, labels assigned to cells in the sketch are propagated to the remaining cells using *k*-nearest neighbor classification. The dotted line indicates the ARI score achieved by clustering the full data using the same Louvain algorithm. Results on datasets zeisel, klein, and saunders are shown in Figure S4.

A common goal in scRNA-seq data analysis is to discover and characterize cell types, typically through clustering methods. The quality of the clustering therefore plays a critical role in biological discovery. The compact size of a geometric or spherical sketch that accurately summarizes the transcriptional heterogeneity in the full data facilitates such downstream analyses. Furthermore, Hie et al. (2019) observed that a more balanced composition of abundant and rare cell types in a geometric sketch allows to better distinguish between cell types compared with a uniform sampling approach. Here, we apply a similar strategy as in Hie et al. (2019) to evaluate the capability of a standard clustering algorithm to distinguish cell types based on our spherical sketch as compared with the geometric sketch. We first cluster the sketches using the graph-based Louvain algorithm (Blondel et al., 2008) and then propagate the labels to the remaining cells by *k*-nearest neighbor classification. We use the Adjusted Rand Index (ARI) (Hubert and Arabie, 1985) to measure the similarity between the inferred clusterings and the ground truth clustering, which is based on the biological cell types taken from the original study. Hie et al. (2019) demonstrated that unsupervised clustering of geometric sketches consistently outperform clusterings of uniformly sampled cells, whereas data-dependent methods *k*-means++ and SRS provide competitive results on only a few instances. In Figures 3 and S4 we show that the more even sampling of the transcriptional landscape by our spherical sketch facilitates the detection of biological cell types. Across datasets and sampling sizes, the clustering of our spherical sketches achieves better or comparable separation of cell types than the clustering of the corresponding geometric sketch. In only three out of thirty-six instances, geometric sketching yielded slightly better median ARI scores. Remarkably, in several cases the clustering of sketches better agrees with the true biological cell types than the clustering based on the full data. This observation is consistent with the assumption of a more balanced composition of cell types in a sketch, but an artifact of the clustering algorithm cannot be excluded, especially in light of the *impossibility theorem for clustering* (Kleinberg, 2003). Note that despite a small variability in Hausdorff distance, the non-deterministic behavior of the Louvain algorithm contributes to the different ARI scores observed in the repeated clustering of spherical sketches.

### Impact of Distance Metrics

Downstream analysis of scRNA-seq such as clustering and trajectory inference relies on a metric that measures the distance between cells in gene expression space. Distance metrics such as Euclidean distance, correlation-based distance, and cosine similarity (adapted as distance) have been proposed as adequate measures of dissimilarity, and its specific choice might depend on assumptions made by computational analysis methods, properties of datasets, and the specific task at hand (Kim et al., 2018, Jaskowiak et al., 2014). Although the Hausdorff distance is defined based on a given metric, geometric sketching ignores the metric space and considers absolute differences in each dimension independently.

**Figure 4:**
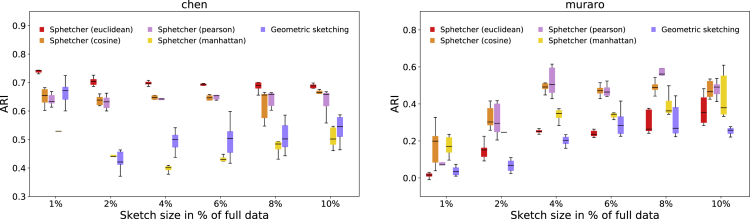
Impact of Distance Metrics on Clustering Performance Although clustering based on spherical sketches computed by Sphetcher using Euclidean distance yields most accurate results on dataset chen, alternative metrics used by Sphetcher lead to higher ARI scores on dataset muraro, illustrating the importance of Sphetcher's flexible optimization scheme. In contrast, geometric sketching does not distinguish different distance metrics and yields overall less accurate clusterings.

Here, we illustrate the flexibility of Sphetcher in optimizing the Hausdorff distance under different distance metrics (see Transparent Methods) and demonstrate that the choice of metric can impact downstream clustering analysis of scRNA-seq data. To this end, we sample a subset of cells from a medium size dataset with complex population structure (*chen*) using Sphetcher with four different metrics: Euclidean, Manhattan, cosine, and Pearson correlation distance. We cluster the four resulting sketches using the same approach as in the previous section and compare the quality of the clusterings with the one obtained from a geometric sketch. Note that the geometric sketching approach proposed in Hie et al. (2019) cannot distinguish different distance metrics. Figure 4 shows that spherical sketches computed by Sphetcher using Euclidean distance as metric in the objective function yield most accurate clusterings of this dataset. Although cosine and Pearson distances have a slightly negative effect on the quality of the clustering, Manhattan distance and geometric sketching yield substantially less accurate clusterings, especially for small sketch sizes.

On dataset *muraro*, geometric sketching again achieves overall lower ARI scores than Sphetcher using different metrics (Figure 4). In contrast to dataset *chen*, however, Euclidean-distance-based sampling does not show any improvement over alternative metrics, illustrating the benefit of Sphetcher's unique ability to take into account different metrics suitable for different tasks.

### Sphetcher Detects Rare Population of Inflammatory Macrophages

Hie et al. (2019) report and experimentally validate the discovery of a rare population of inflammatory macrophages by clustering a geometric sketch of 20,000 cells sampled from a dataset of 254,941 umbilical cord blood cells. In contrast, clustering the full dataset or a uniform subsample did not reveal this rare population of cells, presumably due to their limited visibility among the more abundant inactive macrophages. We repeated the experiment by clustering our spherical sketch of same size (20,000 cells) obtained after prior grid sampling (Sphetcher-H, Transparent Methods) using the Louvain community detection algorithm. As expected, we were also able to discover a similar cluster of inflammatory macrophages based on the same set of marker genes CD74, HLA-DRA, B2M, and JUNB (AUROC >0.88, Transparent Methods).

### Fairness Incorporates Time Points in Trajectory Reconstruction

**Figure 5:**
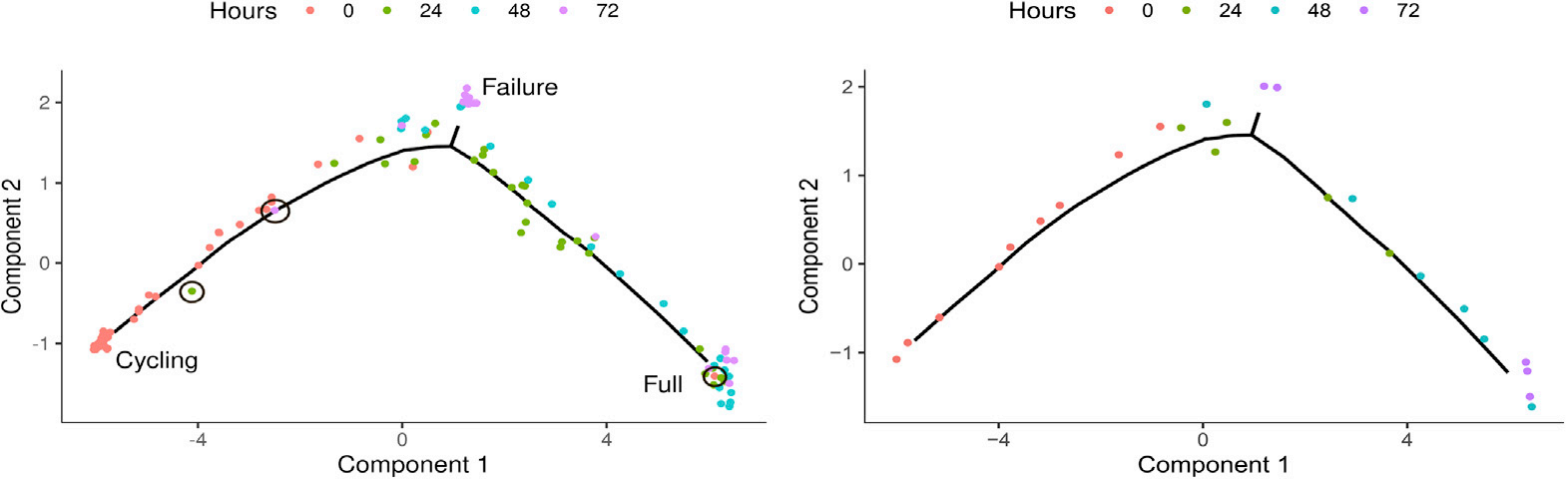
Single-Cell Trajectories of HSMM Differentiation Single-cell trajectories of HSMM differentiation as reconstructed by Monocle 2 from the full data (left) and from Sphetcher's spherical sketch with fairness constraints (right) consistently describe progression through differentiation. Cells for which inferred pseudotime and collection time point disagree are marked with a black circle and were automatically removed as “outlier” cells by Sphetcher. See also Figures S5–S7.

In time series studies of gene expression, single cells are typically collected at different (known) time points. In this section, we illustrate how fairness aspects can be used to incorporate this additional information into the construction of a spherical sketch. To compare the gene expression dynamics of human skeletal muscle myoblast (HSMM) differentiation with the reprogramming of fibroblasts to myotubes, in Cacchiarelli et al. (2018), single cells were sampled every 24 h post-induction of myoblast differentiation, between 0 and 72 h. Consistent with the original publication, we reconstruct the single-cell trajectory of HSMM differentiation using Monocle 2 (Qiu et al., 2017), ignoring the information on the collection time point of cells. Figure 5 (left) shows the resulting trajectory, in which cells are initially in a cycling state and either fully progress to contractile myotubes or fail to differentiate. Cells are colored by the four different time points. For marked cells (black circle) the inferred pseudotime, i.e. their level of progression through differentiation, and the actual time they were collected, disagree. Even though cells do not always progress through the process of differentiation in a synchronous manner, the presence of fully differentiated cells at time point 0, for example, is most likely an artifact caused by noise in the single-cell measurements.

We sought to automatically detect and remove cells for which the collection time point disagrees with their transcriptomic state through a constrained sketching approach. Instead of imposing a hard constraint that removes “outlier” cells, we let our sketching algorithm decide if cells at different time points are necessary to evenly represent the global transcriptional space. Because our fairness-inspired model imposes covering constraints that require a certain number of cells to be sampled from each time point (Transparent Methods), a fair sampling of cells will implicitly discourage the selection of outlier cells that lie close to cells in a similar state but which have been collected at different time points.

We compare the trajectories computed by Monocle 2 from the geometric sketch, our (unconstrained) spherical sketch, and our fairness-inspired spherical sketch that picks at least four cells from each time point. On all sketches, the overall structure of the inferred trajectory agrees with the trajectory computed from the full data (Figures 5 (right) and S5–S7). However, although outlier cells are included in both the geometric sketch (8 out of 8 trials, Figure S6) and the unconstrained spherical sketch (2 out of 8 trials, Figure S7), Sphetcher under fairness constraints decides to not use outlier cells to represent the transcriptional space. Fairness encourages Sphetcher, for example, to not include fully differentiated cells from time point 0 into the sketch (Figures 5 (right) and S5). Even more, although constrained Sphetcher includes at least one cell collected at time point 72 in the final state (Full) in Figure 5 and in all trials in Figure S5, unconstrained sketches do not retain any such cell in any but a single trial (Figures S6 and S7).

In addition, we construct gene expression kinetics plots using Monocle 2 for a set of genes assessed in Cacchiarelli et al. (2018). The expression dynamics inferred from our fair spherical sketch appear smoother than those obtained from the full data, and cells in our sketch better fit the interpolated expression (Figure S8).

### Scalability

Here, we demonstrate scalability of our hybrid strategy Sphetcher-H that combines grid sampling with subsequent spherical sketching (Transparent Methods) to large single-cell datasets. In Table 1 we compare the running time of Sphetcher-H with the construction of a geometric sketch (Hie et al., 2019) on the zeiselCNS, saunders, and umbilical cord blood datasets used in previous benchmarks as well as on a dataset (*cao*) comprising two million cells (Cao et al., 2019). On the latter dataset, geometric sketching and Sphetcher-H require in total around 30 min and 16 min of computation, respectively. Remarkably, our naive grid sampling strategy alone is orders of magnitude faster than geometric sketching but achieves competitive Hausdorff distances on the zeiselCNS and saunders datasets (Figure S3).

**Table 1:** Comparison of CPU Time (in Seconds) of Geometric Sketching and Sphetcher-H

Dataset	# Cells	Sphetcher-H			Geometric Sketching
		Grid	Distances	Set Cover	
Cord blood	254,941	1.0	43.0	88.0	23.0
ZeiselCNS	464,713	3.0	153.0	116.0	120.0
Saunders	665,385	5.0	318.0	200.0	201.0
Cao	2,026,641	10.0	600.0	400.0	1869.0

Running times are reported separately for the prior grid sampling, the calculation of pairwise distances, and the computation of a covering of all cells with spheres using a greedy set cover approach (Transparent Methods).

## Discussion

We have introduced Sphetcher, a novel method that computes a small sketch of single-cell datasets that accurately summarizes its transcriptional heterogeneity. Sphetcher utilizes the thresholding technique to efficiently pick representative cells within spheres that better approximate the global geometry than boxes. Furthermore, we provide theoretical justification for its robust performance in practice. Sphetcher is able to accelerate scRNA-seq analyses such as the detection of cell types through clustering or the reconstruction of developmental trajectories. At the same time, it has the ability to shift the focus from a “more data, less algorithm” regime to a “less (but accurate) data, more algorithm” approach. For example, highly accurate yet computationally expensive algorithms such as consensus clustering by SC3 (Kiselev et al., 2017) might become practical again on a spherical sketch computed by Sphetcher from a large-scale dataset. In addition, Sphetcher is sensitive to rare cell types, is flexible in its use of different distance metrics, and allows to use prior categorical information on, e.g., biological cell types or collection time point to guide the selection of cells into a representative sketch.

### Limitations of the Study

In most of the experiments in this study, Sphetcher used Pearson correlation as distance metric and was combined with a specific algorithm for downstream analysis. Even though Louvain community detection and Monocle 2 are widely used for scRNA-seq clustering and the inference of single-cell trajectories, respectively, Sphetcher's underlying model might be less compatible with assumptions made by other algorithms. In particular, Sphetcher's aim to minimize the maximum distance of cells to the sketch according to some metric might conflict with internal preprocessing routines applied by computational scRNA-seq analysis software. This interplay of sketching with respect to a given distance metric and subsequent algorithmic analysis was not systematically addressed in this study.

Furthermore, the incorporation of collection time points of cells in trajectory reconstruction demonstrates proof of principle. Additional experiments are required to fully address the benefits of leveraging prior (partial) knowledge on, e.g., cell types in the selection of representative cells into a sketch.

### Resource Availability

#### Lead Contact

Further information and requests for resources should be directed to and will be fulfilled by the Lead Contact, Stefan Canzar (canzar@genzentrum.lmu.de).

#### Materials Availability

This study did not generate new materials.

#### Data and Code Availability

Sphetcher is available at https://github.com/canzarlab/Sphetcher, where we also make spherical sketches of public, large scRNA-seq dataset available for download.

## Methods

All methods can be found in the accompanying Transparent Methods supplemental file.

## Supplemental Information

Document S1. Transparent Methods and Figures S1–S8
