# Supplementary material for: Sphetcher: Spherical Thresholding Improves Sketching of Single-Cell Transcriptomic Heterogeneity
Source: iScience. 2020 May 4;23(6):101126. doi: 10.1016/j.isci.2020.101126 (PMC7235285; doi:10.1016/j.isci.2020.101126)
Supplement: Document S1. Transparent Methods and Figures S1–S8 [file mmc1.pdf]

iScience, Volume *23*

Supplemental Information

Sphetcher: Spherical Thresholding Improves Sketching of Single-Cell Transcriptomic Heterogeneity

Van Hoan Do, Khaled Elbassioni, and Stefan Canzar

Supplemental Figures

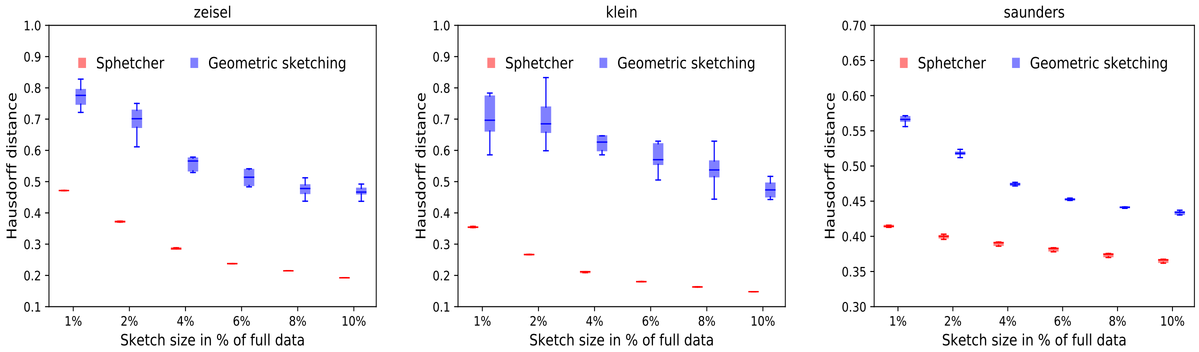

Figure S1. Comparison of Hausdorff distances, related to Figure 2. The spherical sketch computed by Sphetcher exhibits consistently smaller Hausdorff distances to the full dataset than geometric sketching, across datasets and sketch sizes. For each sketch size, the results of 10 random trials are shown.

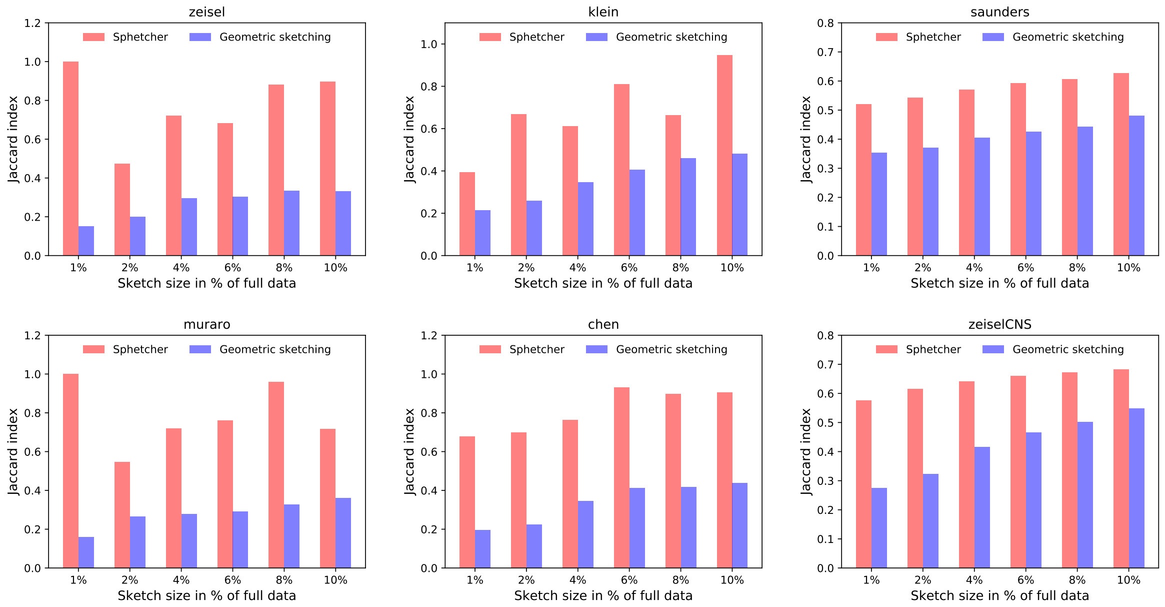

Figure S2. Comparison of Jaccard index, related to Figure 2. We compare the composition of the sketches computed in different random trials. The Jaccard index is computed for all pairs of random trials and the average is taken over all pairs for a given sketch size. The Jaccard index measures the similarity of two sketches by dividing the number of cells that they have in common by the total number of cells contained in either of the sketches. The Jaccard index ranges from 0 to 1, where 0 indicates that the two sketches have no cells in common, while 1 indicates identical sketches. Sphetcher returns highly similar sketches in different random trials, while the set of cells contained in geometric sketches can vary considerably between runs. In addition, these different geometric sketches differ in the quality of representation of the original transcriptomic space (Figure 2 and Figure S1). Note that the similarity of geometric sketches returned in different runs slowly increases with larger sample size, since the algorithm has fewer choices to pick a cell in smaller boxes. In contrast, Sphetcher's random tie breaking between equal-sized sets does not depend on the sample size and thus provides highly stable sketches even for small numbers of cells.

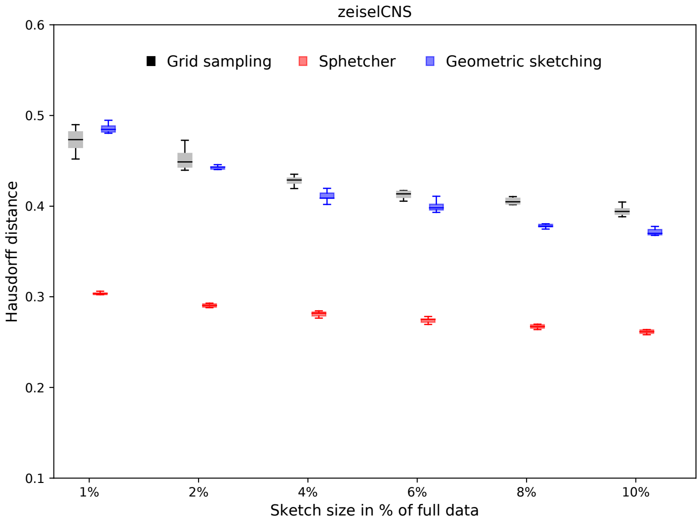

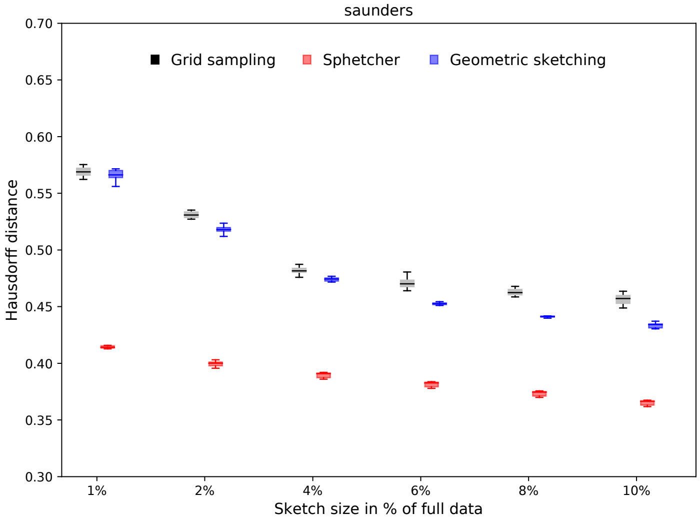

Figure S3. Comparison of Hausdorff distances, related to Figure 2. The naïve grid sampling strategy alone, which is part of our hybrid alternative for very large datasets (Transparent Methods), achieves competitive Hausdorff distances to geometric sketching on datasets zeiselCNS and saunders, especially for small sketch sizes. For each sketch size, the results of 10 random trials are shown.

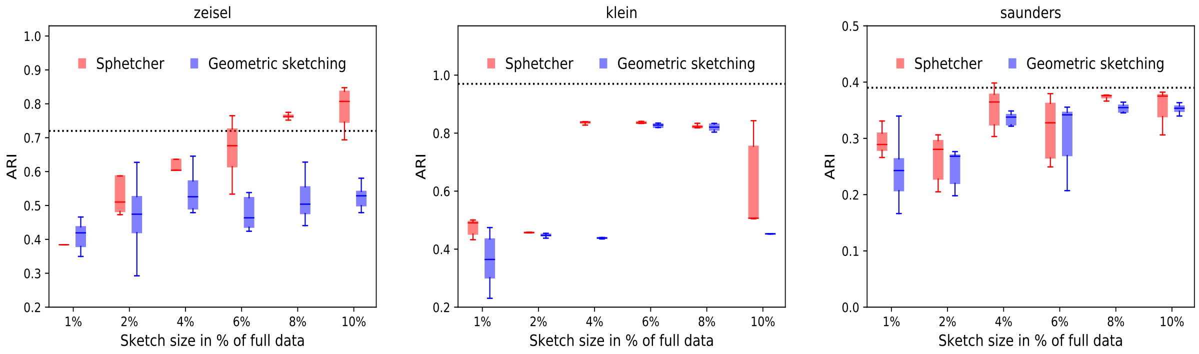

Figure S4. Comparison of sketch based clustering accuracy, related to Figure 3. Louvain clustering of spherical sketches computed by Sphetcher yields more accurate cell clusterings than geometric sketching based clustering. The dotted line indicates the ARI score achieved by clustering the full data using the same Louvain algorithm.

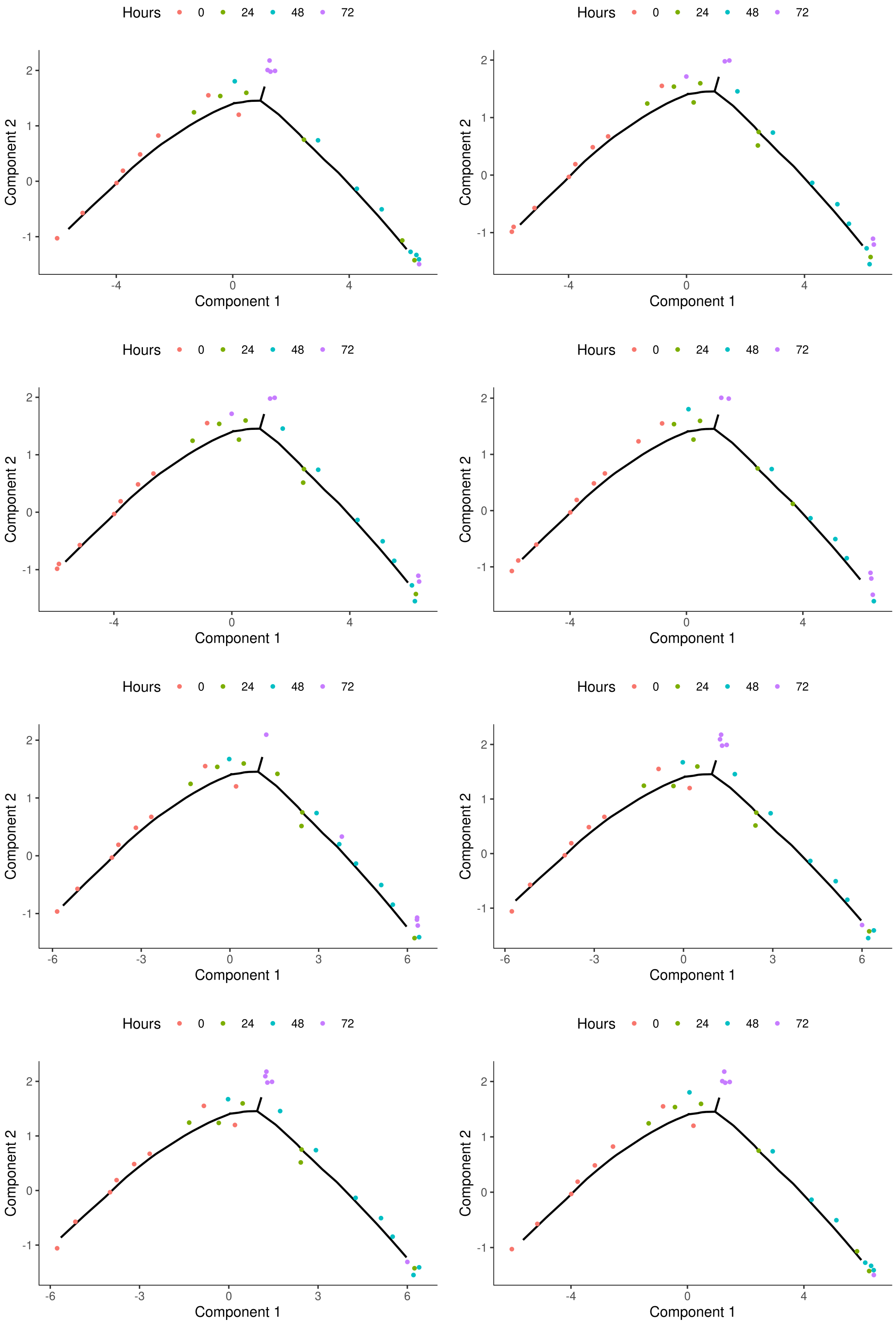

Figure S5. HSMM differentiation trajectories reconstructed by Monocole 2 from Sphetcher's sketch with fairness constraints, related to Figure 5. In 8 trials, Sphetcher did not include 'outlier' cells when its fairness model requires to include at least 4 cells from each time point. For outlier cells inferred pseudotime and actual collection time disagree. At the same time, cells collected at time point 72 in the final state are consistently retained.

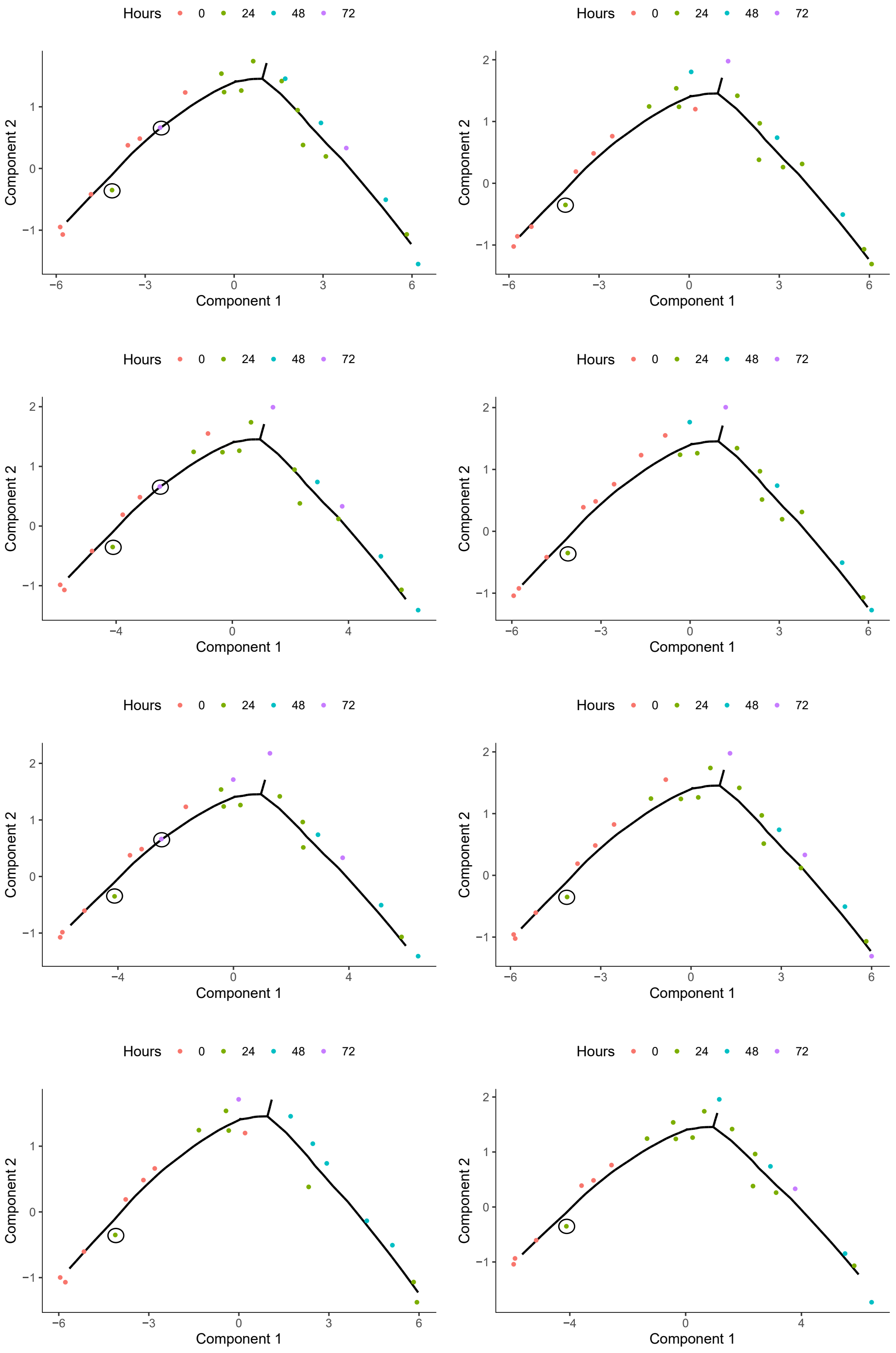

Figure S6. HSMM differentiation trajectories reconstructed by Monocole 2 from geometric sketches, related to Figure 5. In each of 8 trials, geometric sketches included outlier cells (black circles) for which inferred pseudotime and actual collection time disagree. At the same time, in only a single case a cell in final state collected at time point 72 is retained.

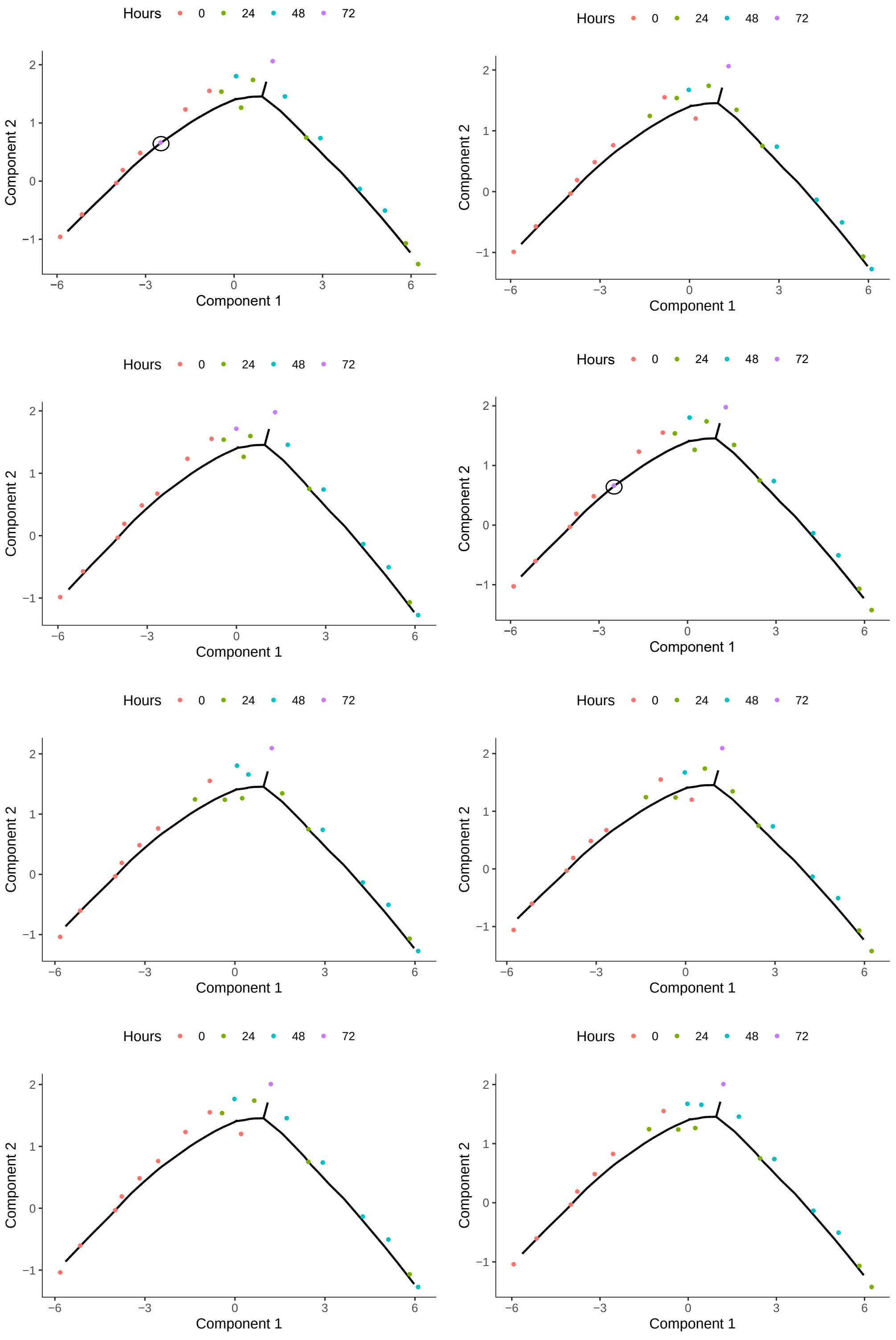

Figure S7. HSMM differentiation trajectories reconstructed by Monocole 2 from Sphetcher's sketch without fairness constraints, related to Figure 5. In 2 out of 8 trials, spherical sketches included outlier cells (black circles) for which inferred pseudotime and actual collection time disagree. At the same time, cells in final state collected at time point 72 are lost in each trial.

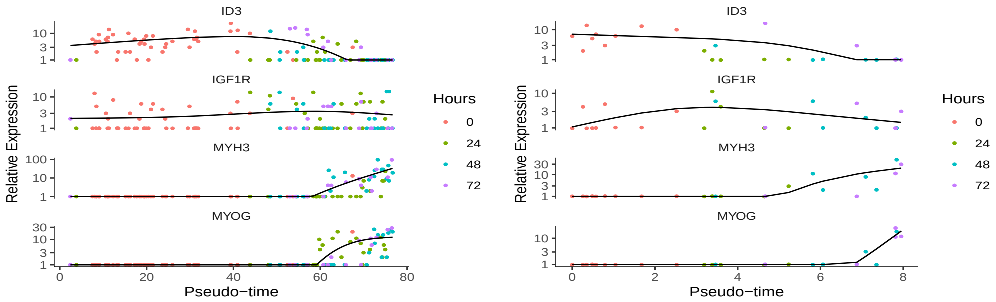

Figure S8. Gene expression dynamics, related to Figure 5. Expression dynamics along pseudotime were computed by Monocle 2 from full data (left) and from the sketch produced by Sphetcher with fairness constraints (right) for genes ID3, IGF1R, MYH3, and MYOG.

Transparent Methods

Sketching scRNA-seq as k-center problem

Given a large scRNA-seq dataset, we seek to select a subset of cells, a so-called *sketch* (Hie et al. 2019), that evenly represents the geometry of the transcriptional space occupied by the original data. As originally proposed in Hie et al. (2019), we use the *Hausdorff distance* to measure how well the sketch captures the transcriptional heterogeneity in the data. Given n data points $X = \{x_1, x_2, \ldots, x_n\}$ representing the m-dimensional gene expression measurements $x_i \in \mathbb{R}^m$ of n individual cells, and a metric d that measures the dissimilarity between pairs of cells, the Hausdorff distances between a sketch $X_S \subseteq X$ and the full dataset is given by:

$$d_H(X_S, X) = \max_{x \in X} \left\{ \min_{y \in X_S} d(x, y) \right\} \tag{1}$$

A sketch achieves a small Hausdorff distance if it includes for every cell in the original dataset a cell that is close to it in gene expression space. Finding a best sketch of size k, i.e. a sketch that minimizes the Hausdorff distance is known as the metric k-center problem in the combinatorial optimization literature. It is known to be NP-hard, but a solution with Hausdorff distance at most 2 times the optimal distance can be found by a simple greedy strategy: In each iteration, pick the point farthest away from the current set of centers and add it as a new center. Although this greedy approach has time complexity $O(nk)$, it does not scale efficiently to large scRNA-seq datasets that require a larger number of cells k to be accurately represented.

A thresholding algorithm

To find a sketch of size k with small Hausdorff distance (1) to a single-cell dataset, we employ the *thresholding* technique that was originally proposed for the design of approximation algorithms for bottleneck problems (Hochbaum & Shmoys 1986). In essence, we are guessing the optimal distance in (1) and for every guess L try to find a feasible solution, that is, a subset of cells of cardinality at most k such that spheres of radius L centered at cells in the subset cover all remaining cells. Then the smallest L^* for which such a feasible sketch exists denotes the optimal solution. We model the problem of finding the smallest set of cells such that the maximal distances from any other cell to the subset is at most a given threshold L as a set cover problem, $\textsc{SetCover}_X(L)$: Given a universe $\mathcal{U} = X$ of n data points, we build a collection $\mathcal{S} = \{S_1, S_2, \ldots, S_n\}$ of n subsets of $\mathcal{U}$ by including in each set S_i all points that lie within distance of L from x_i, i.e. $S_i = \{x_j \mid d(x_i, x_j) \leq L\}$. Then the minimum number of sets in $\mathcal{S}$ that cover every element of the universe corresponds to a smallest subset of points covering all remaining points with spheres of radius L.

A widely used algorithm for the set cover problem is based on a greedy strategy (Johnson 1974): Starting from an empty set, in each iteration pick the set in $\mathcal{S}$ that covers the largest number of elements yet uncovered and add it to the solution. The greedy algorithm is guaranteed to find a cover which is within a logarithmic factor of the optimal solution (Johnson 1974). Moreover, it has been observed across a wide range of instances that the greedy algorithm produces solutions close to the optimum. A direct implementation of the greedy algorithm, however, scales poorly to large scRNA-seq datasets. We therefore employ the disk-friendly greedy (DFG) algorithm developed in Cormode et al. (2010) for very large datasets. It achieves a dramatic performance improvement over the standard greedy algorithm by applying a geometric scale bucketing approximation. Furthermore, the DFG algorithm runs in linear time with respect to the total size of candidate sets, i.e in $O(\sum_i |S_i|)$, while guaranteeing to output a set cover which is within a logarithmic factor of the optimum. More precisely, the algorithm allows to choose a parameter p that represents a trade-off

between the running time (which is $O((1 + \frac{1}{p-1}) \sum_i |S_i|))$ and the approximation ratio (which is $1 + p \ln n$). The complete algorithm is summarized in Algorithm 1. Let us denote by GREEDY(L) the set cover returned by the greedy algorithm when applied to sets $S_i = \{x_j \mid d(x_i, x_j) \leq L\}$, and let $\widetilde{L}(k) := \min\{L \mid \text{GREEDY}(L) \text{ has size at most } k\}$ which can be found by a logarithmic number of calls to the greedy algorithm via binary search: If GREEDY(L) is at most k, we decrease the threshold, otherwise we increase it (halving the length of the search interval in both cases), until the radius L lies in an interval of size at most ε.

Algorithm 1: Sphetcher

Input: Dataset $X = \{x_1, \ldots, x_n\} \subset \mathbb{R}^m$, size of the sketch k, and precision ε.
Initialization: $L_{\min} = 0$, $L_{\max} = \max_{i,j} d(x_i, x_j)$.
while $L_{max} - L_{min} > \varepsilon$ **do**
  $L \leftarrow (L_{\min} + L_{\max})/2$
  Solve SETCOVER$_X(L)$ using the DFG algorithm.
  if $|\text{GREEDY}(L)| \leq k$ **then**
    $L_{\max} \leftarrow L$
  else
    $L_{\min} \leftarrow L$
  end
end
Output: $X_S = \{x_i | S_i \in \text{GREEDY}(L)\}$.

If we are willing to increase the size of X_S by a logarithmic factor, Algorithm 1 is guaranteed to return a sketch with optimal Hausdorff distance.

Theorem 1. *Let L^* be the optimal distance in (1) for $|X_S| = k$. If we run the thresholding approach for $|X_S| = k \ln(n)$, then the solution we obtain has Hausdorff distance at most L^*. In other words, $\widetilde{L}(k \ln(n)) \leq L^*$.*

Proof. By definition of L^*, SETCOVER$_X(L^*)$ has size at most k. Thus, by the known approximation factor of the greedy algorithm, GREEDY(L^*) has size at most $k \ln(n)$, which implies by the definition of $\widetilde{L}(k \ln(n))$ that $\widetilde{L}(k \ln(n)) \leq L^*$. □

Grid sampling with guarantees

For datasets much larger than 100,000 cells, we apply a hybrid strategy to reduce the computational cost of determining the neighborhood of each point in Algorithm 1. To this end, we divide the space into equal-sized boxes from which we pick one point at random. In contrast to geometric sketching, we do not attempt to optimally define boxes in each dimension, but leave it to the subsequent thresholding algorithm to properly cover the space by spheres. In fact, we show that if we carefully choose the applied threshold taking into account the size of the grid, our hybrid sampling strategy increases the Hausdorff distance by at most a factor of $(1 + \varepsilon)$, where $\varepsilon > 0$ controls the size of the grid.

Let SETCOVER$_X(L, Z)$ denote an optimal set covering all the points in X with spheres of radius L whose centers are chosen from $Z \subseteq X$. Let GREEDY(L, Z) denote the set obtained by the greedy algorithm described above covering all the points in X with spheres of radius L whose centers are chosen from $Z \subseteq X$. We know that $|\text{GREEDY}(L, Z)| \leq |\text{SETCOVER}_X(L, Z)| \ln(n)$, where $n = |X|$. Let $L_{\min}$ be the minimum distance between two points in X and $L_{\max}$ be the maximum distance

between two points in X. Let I be the smallest integer such that $(1+\varepsilon)^I L_{\min} \geq L_{\max}$. Our hybrid algorithm that carefully combines grid sampling with the thresholding approach is given in Algorithm 2 (Sphetcher-H).

Algorithm 2: Sphetcher-H

1 **Input**: Dataset $X = \{x_1, \ldots, x_n\} \subset \mathbb{R}^m$, size of the sketch k, and $\varepsilon > 0$.
2 **Initialization**: $L_{\min} = \min_{i,j} d(x_i, x_j)$, an integer I as defined before.
3 **for** i = 0,..., I **do**
4     $L \leftarrow (1+\varepsilon)^i L_{\min}$
5   Partition the space into a uniform grid G(L) of size $\varepsilon L/\sqrt{m}$
6   Let $Z(L) \subseteq X$ be the set obtained by choosing one point in each non-empty cell
7     $Y(L) \leftarrow \textsc{Greedy}\big((1+(1+\varepsilon)\varepsilon)L, Z(L)\big)$
8 **end**
9 **Output**: $Y(\hat{L}(k))$, where $\hat{L}(k) = \min\{L : |Y(L)| \leq k\}$.

The following theorem limits the increase in Hausdorff distance through Sphetcher-H by at most a factor of $(1+\varepsilon)$.

Theorem 2. *Let L^* be the Hausdorff distance $d_H(X_S, X)$ between X and an optimal set X_S of size k, then $d_H(Y(\hat{L}(k\ln(n))), X) \leq (1+\varepsilon)L^*$.*

Proof. Let L be the distance set in the for loop (Algorithm 2: steps 3 to 7) such that $L^* \leq L < (1+\varepsilon)L^*$. By definition of L^*, we know that $|\textsc{SetCover}_X(L^*, X)| \leq k$. So, let us write $\textsc{SetCover}_X(L^*, X) = X_S := \{x_1, ..., x_k\}$. Let $X'_S = \{x'_1, ..., x'_k\} \subseteq Z(L)$ be chosen such that x'_i lies in the same cell of the grid $G(L)$ as x_i. Hence, $d_H(x_i, x'_i) \leq \varepsilon L$ implies that

$$d_H(X_S, X'_S) \leq \varepsilon L < (1+\varepsilon)\varepsilon L^*.$$

Thus for any point $x \in X$, we have

$$d_H(x, X'_S) \leq d_H(x, X_S) + d_H(X_S, X'_S) \leq (1+(1+\varepsilon)\varepsilon)L^* \leq (1+(1+\varepsilon)\varepsilon)L.$$

It follows that $|\textsc{SetCover}_X((1+(1+\varepsilon)\varepsilon)L, Z(L))| \leq k$ and hence,

$$|\textsc{Greedy}((1+(1+\varepsilon)\varepsilon)L, Z(L))| \leq k\ln(n),$$

that is, $|Y(L)| \leq k\ln(n)$. By definition of $\hat{L}$, we have $\hat{L} \leq L < (1+\varepsilon)L^*$. □

Fair sampling

One of the advantages of our model is its flexibility to incorporate fairness aspects. For example, assume we have prior knowledge of (some) of the cell types present in the sample. Cells might have been pre-sorted, and some cell types such as T cell subtypes are well characterized and can be identified based on known markers, without relying on an unsupervised clustering of the data. Furthermore, when reusing scRNA-seq datasets shared through repositories or data archives, the annotation of cell types, i.e. their labels, are typically provided as part of the original study. Similarly, in time series studies of gene expression, cells are collected at different time points which can supervise the sketching algorithm to preferentially select cells for which collection time point and transcriptomic state agree.

Our goal is to use prior categorical information on, e.g., biological cell types or collection time point to guide the selection of cells into a representative sketch, without fully relying on the correctness of cell type labels nor their synchronous progression through biological processes. We incorporate prior categorical information as *covering constraints* into our model: We seek to select a subset of cells that represent the geometric space of the original data according to (1) but at the same time contain at least a given number of representatives from each class. More formally, let $X_1, X_2, \ldots, X_m \subseteq X$ denote known clusters that do not necessarily partition the whole dataset X, we want to sample k cells that contain at least $l_i \in \mathbb{N}^+$ cells from each X_i, for all $i = 1, 2, \ldots, m$, while minimizing the Hausdorff distance of the sketch to the original dataset. This generalization of the k-center problem is similar to the *colorful k-center* problem, which does not require to include class members into the sketch but instead a certain number of elements from each class need to be covered by spheres around selected centers. For the colorful k-center problem a constant approximation in the Euclidean plane was recently introduced (Bandyapadhyay et al. 2019). In Anegg et al. (2020), the authors study a variant of this problem in which classes are allowed to overlap. Neither of the proposed algorithms is directly applicable to scRNA-seq data, due to low-dimensionality assumptions or the use of the ellipsoid method, respectively.

If $l_i = 1$, for all $i = 1, \ldots, m$, we have hitting set constraints $X_S \cap X_i \neq \emptyset$, $i = 1, \ldots, m$, which can be modeled as m additional elements in the universe of our set cover formulation of the problem. Given a threshold L, the corresponding set cover problem $(\mathcal{U}, \mathcal{S})$ is $\mathcal{U} = \{x_1, \ldots, x_n, X_1, \ldots, X_m\}$ and $\mathcal{S} = \{S_1, S_2, \ldots, S_n\}$ with $S_i = [x_i] \cup \{X_j \mid x_i \in X_j\}$. Here $[x_i]$ contains x_i and its neighbors within distance L. Picking a set S_i into our set cover solution now does not only cover all cells within distance L of x_i, but x_i also hits all clusters $\{X_j \mid x_i \in X_j\}$. Having cast the constrained sampling problem as an instance of our thresholding framework, we solve it by the same algorithm (Algorithm 1). For general $l_i \in \mathbb{N}^+$, we simply partition X_i into l_i parts and apply the above approach, which however is no longer guaranteed to obtain the optimal Hausdorff distance.

Set cover under perturbation

This section provides the theoretical insight for the practical performance of the greedy set cover approach and its robustness to noise present in, e.g., scRNA-seq data. In step 5 of Algorithm 1 we need to construct the neighborhood for every point x_i that contains all points within a given distance threshold. Due to noise, the true distances will be slightly perturbed and yield imprecise estimates of neighborhoods. Since an instance to our set cover formulation contains a set for the neighborhood of each point, error-prone neighborhoods will affect our (greedy) search for the set with the largest number of uncovered elements. Here, we show that as long as we are able to pick a set with large enough number of uncovered elements, we can essentially preserve the approximation guarantee. More precisely, denote by C_t the set of elements covered *after* t iterations of the greedy search ($C_0 = \emptyset$). Assume that in each iteration t, errors in the distances prevent us from finding the set S_t^* with the maximum value of $|S_i \backslash C_{t-1}|$, but instead we select a set S_t such that $E(|S_t \backslash C_{t-1}|) \geq c \max_i |S_i \backslash C_{t-1}|$ for some constant c, where $E(X)$ denotes the expected value of random variable X. We show that with high probability, we will find a set cover within $2 \ln(n)/c$ the size of an optimal solution, which differs only by a constant factor from the approximation guarantee of the (precise) greedy algorithm. Note that inapproximability results (Slavík 1997) show that the greedy algorithm is essentially the best-possible polynomial time approximation algorithm for set cover up to lower order terms. Let $\mathcal{U}$ be the whole set of elements of size n. We have the following theorem.

Theorem 3. *If an iterative algorithm always chooses a set S_t to add to the current solution with*

$$E(|S_t \backslash C_{t-1}| \; \big| C_{t-1}) \geq c \max_i |S_i \backslash C_{t-1}|,$$

for $c \leq 1$, then with (high) probability $1 - \frac{1}{n}$ it returns a set cover that is larger than the optimum set cover by a factor of at most $2\ln(n)/c$.

Proof. Let the number of sets in the optimal solution be σ. We know that at each iteration there is some set that covers at least $|\mathcal{U} \backslash C_t|/\sigma$ new elements. It follows that

$$E(|\mathcal{U} \backslash C_{t+1}| \; \big| C_t) = |\mathcal{U} \backslash C_t| - E(|S_{t+1} \backslash C_t| \; \big| C_t) \leq |\mathcal{U} \backslash C_t| - c \max_i |S_i \backslash C_t| \leq \Big(1 - \frac{c}{\sigma}\Big) |\mathcal{U} \backslash C_t|.$$

Now taking the expectation over all possibilities for C_t we get

$$E(|\mathcal{U} \backslash C_{t+1}|) \leq \Big(1 - \frac{c}{\sigma}\Big) E(|\mathcal{U} \backslash C_t|),$$

and iterating we end up with

$$E(|\mathcal{U} \backslash C_t|) \leq |\mathcal{U}| \Big(1 - \frac{c}{\delta}\Big)^t \leq n e^{-tc/\sigma}.$$

Setting $t = 2\sigma \ln(n)/c$ implies that $E(|\mathcal{U} \backslash C_t|) \leq \frac{1}{n}$, and hence by Markov's Inequality:

$$\Pr(|\mathcal{U} \backslash C_t| \geq 1) \leq E(|\mathcal{U} \backslash C_t|) \leq \frac{1}{n}.$$

Thus, with probability at least $1 - \frac{1}{n}$, the sets we selected form a set cover. □

Benchmarks

Sphetcher

We have implemented Algorithms 1 and 2 along with a fair sampling option in software tool Sphetcher in C++. We applied our hybrid strategy Sphetcher-H (Algorithm 2) on datasets exceeding 200,000 cells, which included datasets zeiselCNS, saunders, cao as well as the umbilical cord blood cells dataset. Unless stated otherwise, Sphetcher uses Pearson correlation as distance metric d, and we set the precision $\varepsilon = 10^{-4}$ in Algorithm 1. Note that throughout this work, the size of our spherical sketch denotes the actual number of cells rather than their logarithmic approximation in Theorem 1.

Data and evaluation

All data were uniformly preprocessed by natural log-transformation of gene counts (after adding a pseudo-count of 1) followed by projection to 100 principle components.

We measure how well a sketch represents the original transcriptomic space by the robust Hausdorff distance. Compared to the classical definition of the Hausdorff distance, the robust variant of the distance between a sketch $X_S \subseteq X$ and the full dataset is less sensitive to outliers (Huttenlocher et al. 1993):

$$d_{HK}(X_S, X) = K^{th}_{x \in X} \Big\{ \min_{y \in X_S} d(x, y) \Big\}, \tag{2}$$

where $K^{th}_{x \in X}$ denotes the Kth largest distance to an element in X. Consistent with Hie et al. (2019), we set $K = \lceil 1\text{e}{-4} \times |X| \rceil$ in our experiments.

Marker genes inflammatory macrophages

We computed AUROC for marker genes reported in Hie et al. (2019) separating inflammatory macrophages from remaining macrophages using the Python package provided with the original publication at `https://github.com/brianhie/geosketch`.

References

Anegg, G., Angelidakis, H., Kurpisz, A. & Zenklusen, R. (2020), A technique for obtaining true approximations for k-center with covering constraints, *in* 'Integer Programming and Combinatorial Optimization', Springer International Publishing.

Bandyapadhyay, S., Inamdar, T., Pai, S. & Varadarajan, K. R. (2019), 'A constant approximation for colorful k-center', *CoRR* **abs/1907.08906**.

Cormode, G., Karloff, H. & Wirth, A. (2010), 'Set cover algorithms for very large datasets', *CIKM* pp. 479 – 488.

Hie, B., Cho, H., DeMeo, B., Bryson, B. & Berger, B. (2019), 'Geometric sketching compactly summarizes the single-cell transcriptomic landscape', *Cell Syst.* **8**(6), 483 – 493.e7.

Hochbaum, D. S. & Shmoys, D. B. (1986), 'A unified approach to approximation algorithms for bottleneck problems', *J. ACM* **33**(3), 533 – 550.

Huttenlocher, D. P., Klanderman, G. A. & Rucklidge, W. J. (1993), 'Comparing images using the hausdorff distance', *IEEE Trans. Pattern Anal. Mach. Intell.* **15**(9), 850–863.

Johnson, D. S. (1974), 'Approximation algorithms for combinatorial problems', *J. Comput. Syst. Sci.* **9**(3), 256 – 278.

Slavík, P. (1997), 'A tight analysis of the greedy algorithm for set cover', *Journal of Algorithms* **25**(2), 237 – 254.
